# Mapping Feline Oncology in Portugal: A National Characterization

**DOI:** 10.3390/ani16030364

**Published:** 2026-01-23

**Authors:** Paula Brilhante-Simões, Ricardo Lopes, Leonor Delgado, Augusto Silva, Fernando Pacheco, Ricardo Marcos, Felisbina Queiroga, Justina Prada

**Affiliations:** 1 INNO Veterinary Laboratories, R. Cândido de Sousa 15, 4710-300 Braga, Portugal; paulabrilhante@inno.pt (P.B.-S.); leonordelgado@inno.pt (L.D.); augustosilva@inno.pt (A.S.); 2 Department of Veterinary and Animal Sciences, University Institute of Health Sciences (IUCS), CESPU, 4585-116 Gandra, Portugal; lopes.rmv@gmail.com; 3 Department of Veterinary Sciences, University of Trás-os-Montes e Alto Douro (UTAD), 5000-801 Vila Real, Portugal; fqueirog@utad.pt; 4 CEDIVET Veterinary Laboratories, Lionesa Business Hub, R. Lionesa 446 C24, 4465-671 Leça do Balio, Portugal; 5 UNIPRO-Oral Pathology and Rehabilitation Research Unit, University Institute of Health Sciences (IUCS), CESPU, 4585-116 Gandra, Portugal; 6 Chemistry Research Centre, University of Trás-os-Montes e Alto Douro (UTAD), 5000-801 Vila Real, Portugal; fpacheco@utad.pt; 7 Cytology and Hematology Diagnostic Services, Laboratory of Histology and Embryology, Department of Microscopy, ICBAS-School of Medicine and Biomedical Sciences, University of Porto (U.Porto), Rua de Jorge Viterbo Ferreira, 228, 4050-313 Porto, Portugal; rmarcos@icbas.up.pt; 8 Animal Morphology and Toxicology Team, CIIMAR, Interdisciplinary Centre of Marine and Environmental Research, University of Porto (U.Porto), 4450-208 Matosinhos, Portugal; 9 Animal and Veterinary Research Centre (CECAV), Associate Laboratory for Animal and Veterinary Sciences (AL4AnimalS), University of Trás-os-Montes e Alto Douro (UTAD), 5000-801 Vila Real, Portugal

**Keywords:** cancer surveillance, epidemiology, histopathology, Portuguese cats, veterinary pathology

## Abstract

This retrospective study describes the national histopathology caseload of feline tumours submitted to a Portuguese diagnostic laboratory over a five-year period. A total of 1904 histopathology-confirmed neoplasms were analysed by biological behaviour, anatomical location, and demographic/geographical variables. Malignant tumours predominated (77.4%), whereas 22.6% were benign. Tumours most commonly involved the mammary gland (44.8%) and cutaneous/soft tissues (42.4%), together accounting for 87.2% of cases; all other sites were individually uncommon (≤5.6%). The most frequent malignant tumour types were mammary carcinoma (38.3%), fibrosarcoma (8.0%), squamous cell carcinoma (6.4%), and mast cell tumour (4.8%). Cats with malignant tumours were older than those with benign lesions (*p* < 0.001), and females comprised most submissions (69.3%), largely driven by mammary neoplasia. Multiple, histologically distinct tumours were identified in 8.3% of cats and were more frequent in older females (*p* = 0.001). Domestic Shorthairs comprised the vast majority of cases, and no significant associations were detected between breed (including pure breeds) or geographical location and tumour occurrence or biological behaviour (*p* > 0.05). These findings highlight a sustained predominance of malignant disease in Portuguese cats, concentrated in mammary and cutaneous/soft-tissue sites, supporting a low threshold for biopsy in older cats and systematic mammary screening in females, and continued registry-based surveillance to monitor temporal changes in tumour patterns.

## Simple Summary

Epidemiological studies play a key role in feline oncology by identifying which tumours are most common, in which patients they occur, and where they are most likely to arise. Mapping tumour occurrence in cats helps clinicians prioritise early diagnostic investigation, supports informed clinical decision-making, and contributes to earlier detection of malignant disease. This study evaluated feline tumour biopsies submitted across Portugal over five years. We analysed 1904 histopathology-confirmed neoplasms to describe where tumours arise, how often they are malignant, and the characteristics of most affected cats. Malignant tumours predominated (77.4%). Nearly nine in ten diagnoses involved two sites: the mammary gland (44.8%, predominantly in females) and the cutaneous/soft tissues (42.4%). Tumours of the gastrointestinal tract, oral cavity, eyes, urinary system and other organs were comparatively uncommon. Cats with malignant disease were older than those with benign lesions. Multiple tumours occurred in 8.3% of cats and were more frequent in older females. We found no meaningful patterns by breed nor evidence of geographical clustering within Portugal. For owners and clinicians, these results support prompt assessment of any new mass, particularly in older queens, and systematic palpation of mammary chains. Ongoing national surveillance of tumour patterns will help track changes over time and support better preventive care and outcomes.

## 1. Introduction

Cancer is increasingly acknowledged as a major health concern in companion animals, and in cats, it represents one of the principal causes of disease-related morbidity and mortality. Feline malignant tumours are frequently characterised by aggressive biological behaviour and poor clinical outcomes, leading to shortened survival and limited therapeutic options [1]. Epidemiological studies consistently indicate that cats are more likely than dogs to develop malignant neoplasms, with estimates suggesting approximately a fourfold higher risk of malignancy in felines [2,3,4,5,6,7,8,9,10]. These findings highlight the significance of feline oncology and the need for veterinarians and owners to be aware of the importance of timely recognition, accurate diagnosis and appropriate management.

Over the past five decades, multiple epidemiological investigations have been undertaken worldwide to characterise the occurrence and distribution of feline tumours. Early surveys conducted in the United States [11,12] provided the first systematic descriptions of tumour prevalence in domestic cats. Since then, retrospective and registry-based studies have been reported in a variety of geographical contexts, including Italy [3,13,14], Sweden [15], Japan [16], South Africa [17], Switzerland [18,19], Mexico [20], Korea [6], Russia [21], Croatia [4], Thailand [22], United Kingdom [23] and Portugal [2,24,25]. Although methodologies varied, these studies consistently confirmed the predominance of malignant neoplasms in cats, with mammary carcinomas, fibrosarcomas, squamous cell carcinomas and lymphomas representing the most frequently diagnosed tumours, although with variation in their relative classification across populations [1,2,6,10,11,13,14,16,17,19,20,24,26,27].

In addition to these epidemiological surveys, numerous review articles have synthesised the literature on feline oncology, consolidating knowledge on both common and less frequent tumour types. Early reviews addressed general prevalence [11], central nervous system neoplasms [28,29], oral tumours [30,31,32,33], and gastrointestinal malignancies [7,34], while other publications have explored comparative oncology [1,27,35], infection-associated tumours [36,37] and broader epidemiological perspectives [4,10,32]. Complementing these overviews, a substantial body of work has focused on tumours arising in specific anatomical locations. Feline mammary tumours have been extensively studied across different populations [3,8,38,39,40,41,42,43,44], while cutaneous and soft tissue tumours [3,23,45,46,47,48,49,50,51] have been described in both retrospective series and case-based analyses. Squamous cell carcinoma has been investigated in the oral cavity and other sites [10,14,17,20,52,53,54,55,56,57,58], while fibrosarcomas remain another major focus of research given their frequency and aggressive behaviour [10,19,23,59,60,61,62,63,64]. Lymphomas [22,65,66,67,68,69,70,71], and rarer tumours such as melanomas [46,72,73], ocular neoplasms [74,75,76], peripheral nerve sheath tumours [48,77], feline giant-cell pleomorphic sarcoma [78] or liver neoplasias [79] have also been described. Collectively, this body of work highlights both the centrality of mammary carcinoma, fibrosarcoma, squamous cell carcinoma and lymphoma in feline oncology and the remarkable diversity of tumour types reported across anatomical systems [1,2,6,10,11,13,14,16,17,20,24,25,26,27].

In Portugal, published data on feline oncology remain scarce. The first nationwide survey, restricted to cases diagnosed in 2019, provided valuable baseline information on tumour distribution and malignancy [24], and the Vet-OncoNet project subsequently established a structured registry and confirmed the disproportionately high prevalence of malignant tumours in cats compared with dogs [2]. Given the limited epidemiological information available in previous studies, often constrained by incomplete clinical data and heterogeneous case submissions, an updated and comprehensive overview of feline oncology is needed to better characterise neoplastic patterns in this species and to extend temporal coverage, provide a more detailed exploration of tumour incidence and distribution, and situate Portuguese data within the broader international context. Such knowledge is of practical relevance to both clinicians and diagnostic laboratories, as it informs early recognition and supports accurate diagnostic decision-making, ultimately contributing to improved patient management and prognostic evaluation. Therefore, the present study aims to address this need by providing an updated epidemiological characterisation of feline neoplasms in Portugal, with consideration of age, sex, breed, anatomical site, and geographical distribution.

## 2. Materials and Methods

### 2.1. Data Collection, Sampling and Diagnostic Procedures

This retrospective analysis is based on 4775 biopsy submissions received over a five-year period by INNO Veterinary Laboratories (Braga, Portugal) from 276 veterinary practices across all Portuguese districts and Autonomous Regions, of which 1904 histopathology-confirmed tumours were retained for study. Each sample was accompanied by a laboratory request form containing essential clinical information, including breed, sex, age, clinical signs or suspected diagnosis, anatomical location, and the analyses requested. Tissues were fixed in 10% neutral buffered formalin, processed routinely, and stained with haematoxylin and eosin (H&E). Histopathological diagnoses were established according to the World Health Organisation (WHO) classification of tumours of domestic animals [80]. All data were retrieved from the Clinidata^®^ (version 5.3.25 Maxdata Software, S.A., Carregado, Portugal) and transferred to Microsoft Excel^®^ (Microsoft, Redmond, WA, USA) sheets.

Non-neoplastic cases (*n* = 2871) were excluded from the study. For this study, cases classified as multiple were defined as additional samples submitted under a separate laboratory request and at a later point relative to the index diagnosis.

### 2.2. Statistical Analysis

For statistical purposes the animal age was classified into seven groups using and adaptation of a previous methodology: kitten (≤1 year old), young (>1 to ≤2 years) old, young adult (>2 to ≤4 years old), adult (>4 to ≤7 years old), mature adult (>7 to ≤10 years old), senior (>10 to ≤15 years old) and geriatric (>15 years old) [81,82].

The samples were grouped for statistical treatment by anatomical location into the following categories: body cavity (mesothelium and mediastinum); cutaneous and soft tissues; musculoskeletal system (bone and joints); oral cavity (tongue, lips, gingiva, palate, and mouth not otherwise specified–MNOS); respiratory system (nasal and lung); mammary gland; reproductive system (uterus and ovaries; and testis); gastrointestinal tract (stomach, intestine, liver, gallbladder, and salivary glands); ocular system; haemolymphatic system (lymph nodes, spleen, and thymus); urinary system (kidney, ureters, bladder, and urethra); and neuroendocrine system (aortic body).

Statistical evaluations were conducted using JMP^®^ version 18.0.0 (SAS Institute, Cary, NC, USA, 1989–2023), DATAtab^®^ (numiqo e.U., Graz, Austria, 2025), and MedCalc^®^ Statistical Software version 20.006 (MedCalc Software Ltd., Ostend, Belgium). Proportional differences were evaluated using the chi-square (χ^2^) test, with Fisher’s exact test applied when more than 20% of expected cell frequencies were below five. Age comparisons between benign and malignant tumours were performed using the Mann–Whitney U test, as age data were not normally distributed (Shapiro–Wilk test, *p* < 0.05). Subsequently, a Scheirer–Ray–Hare test (a non-parametric equivalent of the two-way ANOVA) was applied to evaluate the combined effects of tumour behaviour (benign vs. malignant) and sex on age, based on rank-transformed data. Where applicable, effect sizes were reported as Cramér’s *V* for contingency tables, and the rank-biserial correlation (*r*) for Mann–Whitney tests. Associations between geographical district and tumour occurrence or behaviour were tested using the chi-square test. Statistical significance was set at *p* ≤ 0.05 [83].

## 3. Results

**Figure 1:**
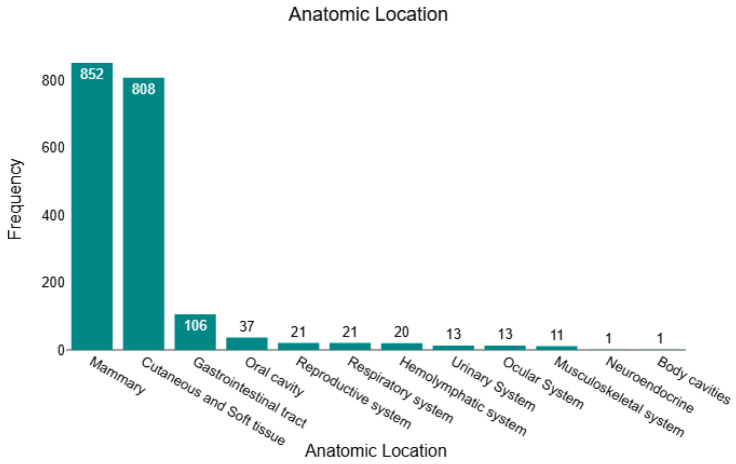
Anatomical distribution of all feline tumours (*n* = 1904).

In this retrospective study, we analysed 1904 histopathology-confirmed tumours, of which 430 (22.6%) were benign, and 1474 (77.4%) were malignant. Across the full cohort, tumours were predominantly located in the mammary gland (*n* = 852; 44.8%) and cutaneous/soft-tissue compartments (*n* = 808; 42.4%), together accounting for 87.2% of all cases. The remaining sites were far less frequent: gastrointestinal tract (*n* = 106; 5.6%), oral cavity (*n* = 37; 1.9%), respiratory system (*n* = 21; 1.1%), reproductive system (*n* = 21; 1.1%), haemolymphatic tissues (*n* = 20; 1.1%), urinary system (*n* = 13; 0.7%), ocular system (*n* = 13; 0.7%), musculoskeletal system (*n* = 11; 0.6%), body cavities (*n* = 1; <0.1%), and neuroendocrine (*n* = 1; <0.1%). This distribution is illustrated in Figure 1, underscoring the dominance of mammary and cutaneous/soft-tissue submissions within the dataset. All sample characteristics are presented in Supplementary Table S1.

### 3.1. Age

From the 1904 animals analysed, age data were available for 1731 (90.9%), whereas 173 (9.1%) requisition forms lacked age specification and were therefore excluded from age-based analyses. The age distribution among these 1731 animals ranged from 3 months (≤1 year) to 21 years, with a median of 10 years (interquartile range [IQR]: 8–12). A total of 2.1% (95% CI: 1.5–2.9; *n* = 36) were ≤1 year, 1.7% (95% CI: 1.2–2.4; *n* = 29) were >1 to ≤2 years, 5.0% (95% CI: 4.1–6.2; *n* = 87) were >2 to ≤4 years, 14.7% (95% CI: 13.1–16.5; *n* = 255) were >4 to ≤7 years, 29.9% (95% CI: 27.8–32.1; *n* = 517) were >7 to ≤10 years, 41.1% (95% CI: 38.8–43.4; *n* = 711) were >10 to ≤15 years, and 5.6% (95% CI: 4.6–6.7; *n* = 96) were >15 years.

**Figure 2:**
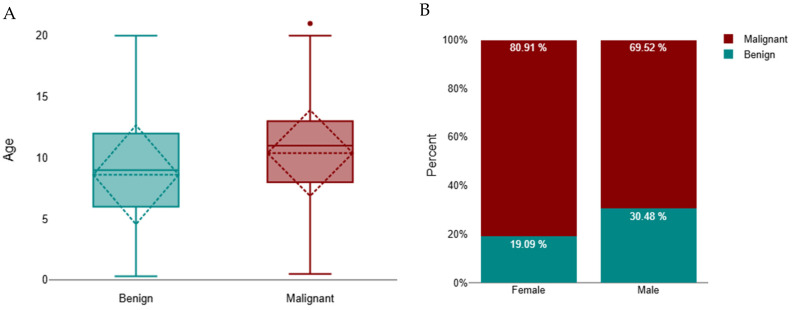
Distribution of benign and malignant tumours in cats. (**A**) Age distribution by tumour type, represented as boxplots showing the median and interquartile range (IQR); whiskers indicate the minimum and maximum values, and outliers are marked as individual points. A significant difference was observed between benign and malignant groups (*p* < 0.001). (**B**) Distribution by sex, showing the proportion of benign and malignant tumours in females and males.

Cats with malignant tumours were significantly older than those with benign tumours (median 11 years, IQR 8–13; *n* = 1341 vs. median 9 years, IQR 6–12; *n* = 390; Mann–Whitney U = 195,572.5, *z* = −7.61, *p* < 0.001; *r* = 0.18) (Figure 2A). A subsequent Scheirer–Ray–Hare test (non-parametric two-way ANOVA) demonstrated that age differed significantly according to tumour behaviour (*p* < 0.001) and that there was a small but significant interaction between tumour behaviour and sex (*p* < 0.001). The main effect of sex alone was not significant (*p* = 0.789), indicating that while overall age did not differ between males and females, the relationship between age and tumour behaviour varied by sex. Specifically, female cats with malignant tumours tended to be older (median 11 years, IQR 8–13) than males with malignant tumours (median 10 years, IQR 7–12), while no substantial sex difference was observed among benign cases (median 9 years, IQR 6–12 for both sexes) (Figure 2B).

### 3.2. Sex

Regarding sexes, 1320 (69.3%) occurred in female cats and 584 (30.7%) in males. Among females, 1068 tumours (80.9%) were malignant and 252 (19.1%) benign, whereas in males, 406 tumours (69.5%) were malignant and 178 (30.5%) benign. Thus, malignant tumours predominated in female cats, while benign tumours were proportionally more frequent in males (Figure 2B). A chi-square test confirmed a significant association between sex and tumour behaviour (χ^2^ = 30.03, *df* = 1, *p* < 0.001). The strength of this association, measured by Cramér’s V (0.18), indicated a small effect size.

### 3.3. Breed

Domestic Shorthair (DSH) cats accounted for the vast majority of cases (*n* = 1755; 92.2%), representing 13 breeds overall. Persian cats were the second-most-represented breed (*n* = 83; 4.4%), followed by Siamese (*n* = 33; 1.7%) and Norwegian Forest (*n* = 9; 0.5%). Other breeds, including Maine Coon (*n* = 5; 0.3%), Scottish Fold (*n* = 4; 0.2%), Ragdoll (*n* = 3; 0.2%), Sphynx (*n* = 3; 0.2%), Russian Blue (*n* = 3; 0.2%), Somali (*n* = 2; 0.1%), British Shorthair (*n* = 2; 0.1%), Turkish Angora (*n* = 1; 0.05%), and Blue Shorthair (*n* = 1; 0.05%), were only sporadically represented. For statistical purposes, DSH cats were excluded from the breed-specific analysis to avoid overrepresentation.

The analysis of breed-related predispositions in tumour behaviour, excluding DSH, did not reveal significant associations. Fisher’s exact test across the 12 pure breeds showed no significant difference in the distribution of benign and malignant tumours (*p* = 0.471). Persians and Siamese were the most frequent pure breeds, while the remaining breeds were sparsely represented, limiting the statistical power of the analysis.

### 3.4. Anatomical Location

Mammary tissues represented the most frequent site overall (44.8%; 95% CI: 42.5–47.0; *n* = 852), with the vast majority being malignant (86.3%; 95% CI: 83.8–88.4; *n* = 735). Cutaneous and soft tissue tumours formed the second largest category (42.4%; 95% CI: 40.2–44.7; *n* = 808), although a comparatively higher proportion of benign lesions was recorded (36.6%; 95% CI: 33.4–40.0; *n* = 296). All gastrointestinal (100%; 95% CI: 96.5–100; *n* = 106), urinary (100%; 95% CI: 77.2–100; *n* = 13), ocular (100%; 95% CI: 77.2–100; *n* = 13), neuroendocrine (100%; 95% CI: 20.7–100; *n* = 1), and body cavity tumours (100%; 95% CI: 20.7–100; *n* = 1) were malignant. Other sites also showed a marked predominance of malignancy, including the respiratory system (100%; 95% CI: 84.5–100; *n* = 21), oral cavity (81.1%; 95% CI: 65.8–90.5; *n* = 30), haemolymphatic tissues (95.0%; 95% CI: 76.4–99.1; *n* = 19), musculoskeletal system (90.9%; 95% CI: 62.3–98.4; *n* = 10), and reproductive system (61.9%; 95% CI: 40.9–79.3; *n* = 13). A chi-square test confirmed a statistically significant association between anatomical location and tumour behaviour (χ^2^ = 174.98, *df* = 11, *p* < 0.001), with the mammary gland (86.3% malignant) and gastrointestinal tract (100% malignant) showing the highest proportions of malignancy. These data are compiled and presented in Table 1.

Age and sex patterns varied by anatomical site. Mammary tumours occurred overwhelmingly in females (96.6%; median 11 years, IQR 8–13), with males rarely affected (3.4%; median 11 years, IQR 9–13). Cutaneous and soft-tissue tumours showed near parity by sex (47.2% female; 52.9% male) and similar ages (females: median 10 years, IQR 7–12; males: median 9 years, IQR 7–12). Gastrointestinal tumours affected both sexes (45.3% female; 54.7% male) at a comparable age (females: median 10 years, IQR 8–13; males: median 11 years, IQR 8–12). Oral cavity tumours tended to present in older animals (females: median 12.5 years, IQR 10–15; males: median 12 years, IQR 9–15) and were more frequent in females (54.1%). Respiratory, urinary, and ocular tumours were male-biased (respiratory 76.2% male; urinary 61.5% male; ocular 84.6% male), with urinary cases occurring at older ages (males: median 13 years, IQR 12–15; females: median 11 years, IQR 5–14) and ocular cases in comparatively younger animals (males: median 7.5 years, IQR 4–9; females: median 9 years, IQR 7–11). Haemolymphatic tumours also showed a male predominance (60% male; median 7.5 years, IQR 6–12), whereas reproductive tumours were, as expected, largely female (95.2%; median 10 years, IQR 7–13). Descriptive statistics by site are presented in Table 2.

### 3.5. Multiple Neoplasia

**Figure 3:**
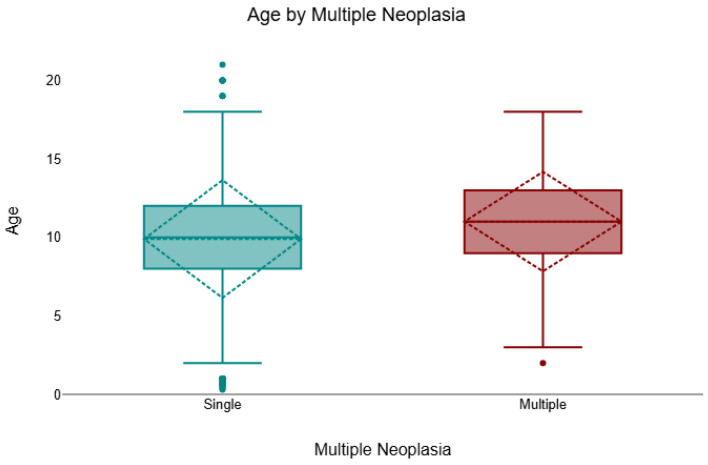
Age distribution by tumour multiplicity (single vs. multiple), shown as boxplots indicating the median and interquartile range (IQR); whiskers denote the minimum and maximum values and outliers are plotted as individual points. Across cases with age available (*n* = 1731/1904), felines with multiple neoplasms were significantly older than those with a single neoplasm (median 11 years, IQR 9–13 vs. 10 years, IQR 8–12; Mann–Whitney U = 195,572.5, *p* = 0.001, *r* = 0.08).

Across the cohort, 8.3% (157/1904) of cases presented multiple neoplasms of different histological types, and 91.8% (1747/1904) were single. Multiple lesions were more frequent in females (132/1320; 10.0%) than in males (25/584; 4.3%). A chi-square test confirmed a significant association between sex and multiplicity (χ^2^ = 17.50, *df* = 1, *p* < 0.001, *V* = 0.13). In the age-available subset (*n* = 1731), animals with multiple neoplasms were significantly older than those with single lesions (Mann–Whitney U test, *p* = 0.001, *r* = 0.08). The median age of cats with multiple tumours was 11 years (IQR 9–13), compared with 10 years (IQR 8–12) for those with single lesions (Figure 3).

### 3.6. Geographical Location

To assess spatial heterogeneity, case counts were aggregated at three administrative scales (e.g., NUTS2–Nomenclature of Territorial Units for Statistics, level 2, district, municipality) [84,85,86,87,88] and evaluated using chi-square tests of independence. No statistically significant association was detected between the occurrence of neoplasia and any geographical partition (*p* > 0.05), both when considering all tumours collectively and when stratifying by tumour behaviour (benign vs. malignant). Accordingly, geographical location was not retained as an explanatory factor in subsequent analyses. The sampling frame encompassed submissions from 276 veterinary practices distributed across districts of mainland Portugal (and the autonomous regions), supporting the representativeness of this null spatial finding.

## 4. Discussion

Our retrospective survey revealed that malignant neoplasms predominated, accounting for 77.4% of cases. This high malignancy rate is broadly consistent with other recent Portuguese data: a nationwide study reported 74.7% malignant tumours [24], and the Vet-OncoNet database reported 78.7% [2]. It also aligns with global reviews highlighting the aggressive nature of feline tumours [1]. In parallel with this malignancy profile, mammary and cutaneous/soft-tissue lesions were vastly overrepresented in our cohort (44.8% and 42.4% of submissions, respectively), accounting for 87.2% of all cases. By contrast, gastrointestinal, ocular and urinary tumours were rare (<7% combined), mirroring established anatomical patterns described in cats [3,14]. Taken together, these findings indicate that histopathology submissions in Portugal remain heavily weighted toward a small number of high-burden sites, particularly mammary gland and cutaneous/soft tissue.

Age and sex distributions further reinforce these epidemiological trends. Cats with malignant tumours were significantly older than those with benign lesions (*p* < 0.001); overall, the median age of neoplastic cases in our cohort was 10 years (IQR 8–12), consistent with the cumulative nature of cancer risk reported elsewhere [1,19]. Although female cats tended to present at older ages than males, this sex imbalance was largely driven by mammary disease: virtually all mammary tumours occurred in queens (96.6% female), in agreement with studies showing that females carry the greatest risk of feline mammary neoplasia [3]. This marked predominance is strongly influenced by sex hormones, as intact queens and those spayed later in life have a significantly increased risk of developing mammary tumours due to prolonged exposure to endogenous oestrogens and progesterone. In addition, the administration of exogenous progestogens has also been consistently identified as an important risk factor [43,89]. Importantly, aside from mammary neoplasia, overall sex differences in tumour behaviour were negligible [19].

In contrast to age and sex, breed and geographic location had little impact on tumour behaviour. Domestic shorthairs overwhelmingly dominated the sample (>92%, as in previous Portuguese data), which likely reflects local population structure rather than a genetic predisposition [14,24]. Consistently, no pure breed showed a higher malignancy risk and no regional “hot-spots” were identified (i.e., tumour incidence did not differ by district or region), mirroring prior reports that an animal’s residence has minimal influence on cancer risk in cats [2,24]. Our findings largely corroborate previous literature.

When contextualised internationally, the predominance of malignant tumours in our series (roughly three-quarters of submissions) is in line with European reports [4,14] and North American registry data [1,11]. Likewise, the dominance of mammary and cutaneous/soft tissue neoplasms is well documented across regions. The proportion of mammary tumours in our cohort (44.8%) closely matches that reported in Portuguese data (43.6% [24]). It exceeds that in some international cohorts (e.g., 37% in Thailand [22] and 40% in the United Kingdom series [23]). This high proportion of mammary tumours in female cats, previously described in our country and also observed in our study, is most likely related to the high number of intact females [89]. However, because reproductive status was not available for our cases, we could not directly test this association. In cutaneous/soft-tissue lesions, fibrosarcoma and other mesenchymal skin cancers accounted for the majority of cases, consistent with global surveys [14,19,51]. In cats, the high prevalence of fibrosarcoma has been widely linked to injection-site sarcomas, particularly associated with vaccination and other injectable products. Chronic local inflammation is thought to play a key role in tumour development in susceptible individuals. Although not all feline fibrosarcomas are vaccine-associated, this well-recognised phenomenon may partly explain the high frequency of fibrosarcoma observed in epidemiological studies [51,90].

In contrast to some studies, which found more cutaneous than mammary cases [6,14], our series had an unusually high mammary: skin ratio, possibly reflecting referral bias or evolving trends. Importantly, our anatomical distribution (with very few gastrointestinal, ocular or urinary tumours) echoes the “classic” picture of feline oncology established by multiple studies [1,32].

Our demographic findings also align with prior work [2,19,23,25]. Older cats were much more likely to harbour malignant tumours, consistent with Graf et al. (2016) and other studies noting increased tumour frequency in middle-aged and senior cats [2,4,6,19,20]. Multiple tumour-bearing individuals were predominantly older, as seen elsewhere. The marked association of mammary cancer with older queens is consistent with Italian, Portuguese, and American series, emphasising that females are the primary affected group for feline mammary carcinoma [3,44,91]. Meanwhile, the absence of a strong sex bias outside the mammary gland is consistent with Vet-OncoNet data [2]. Notably, comparison with previous Portuguese data suggests temporal continuity: the first national survey (752 cases) documented a 74.7% malignancy rate and 43.6% of tumours in the mammary gland [24], and our cohort, collected over a broader time span, identified a similarly high malignancy rate and a nearly identical mammary-tumour proportion. This stability implies that, in Portugal, histopathology caseloads remain consistently weighted toward aggressive cancers.

At the diagnostic level, our results reinforce the consensus that mammary carcinoma, fibrosarcoma, squamous cell carcinoma and lymphoma dominate feline neoplasia [1,2,6,10,14,16,17,19,20,24]. In parallel, the rarity of other sites (e.g., oral cavity, eye, urinary tract) is consistent with reports from diverse regions [4,7,14,32,58,75]. The weak breed agreement in our dataset is also unsurprising: Soares et al. (2021) [24] and Vet-OncoNet [2] similarly found no clear breed–tumour associations in cats, and the null effect of geography mirrors earlier observations that feline cancer incidence is fairly uniform across Portuguese districts [2]. Overall, our data extend the national time horizon while supporting the global epidemiological pattern outlined by Cannon et al. (2015) [1], Cray et al. (2020) [32] and others [2,6,10,14,17,19,20,24].

These patterns have clear clinical implications. Given the high prevalence of malignancy in cats, veterinarians should maintain a low threshold for cytology and/or biopsies of suspicious masses, particularly in older patients and female cats [1]. Routine screening of feline mammary glands and comprehensive dermatologic examination are especially important given the burden of tumours in these sites. The data also underline the value of integrated diagnostic pathways, for instance, pursuing histopathology when cytology is inconclusive, since some fibrous or mixed lesions may be missed on cytology alone [24]. Awareness of these epidemiological trends may improve case management: diagnostic laboratories should recognise that a new skin lesion in an elderly queen is statistically more likely to be a malignant fibrosarcoma or squamous cell carcinoma [2,4,14,16,92]. In practice, clinicians and pathologists can use this information to prioritise advanced work-ups (e.g., immunohistochemistry or molecular assays) for equivocal cases and to counsel owners regarding the likely prognosis. Ultimately, early detection and definitive histological diagnosis remain central to treatment planning and improved outcomes in feline oncology [24].

We acknowledge the limitations of this study. Clinical information (treatment, survival) was often sparse or absent on submission forms, precluding outcomes analyses. In addition, other epidemiologically relevant variables, such as detailed anatomical sublocation of cutaneous and mammary tumours, reproductive status, and the use of exogenous progestagens, were not consistently reported and therefore could not be analysed. Our data also derive from a single commercial pathology service; although it serves all Portuguese districts, it may not encompass every case nationwide. These limitations are typical of veterinary cancer registries [19,25] and underscore the need for more systematic, prospective studies. Furthermore, an inherent selection bias is associated with histopathology-based datasets, as tumours frequently diagnosed by cytology alone, such as lymphoma, may be underrepresented when histopathological confirmation is not pursued in clinical practice. Therefore, the relative frequencies reported here should be interpreted as reflecting tumours submitted for histopathological evaluation rather than the true prevalence of feline neoplasms in the general population. Nonetheless, the large sample size and geographic breadth of our dataset mitigate some biases and provide a robust cross-section of feline oncology in Portugal.

## 5. Conclusions

This nationwide histopathology survey shows a sustained predominance of malignant neoplasms in Portuguese cats (77.4%), with the burden concentrated in the mammary gland (44.8% of all tumours; 86.3% malignant within site) and cutaneous/soft tissues (42.4%), patterns largely presented by older animals and, for mammary neoplasia, overwhelmingly by queens. These trends, together with the rarity of tumours in other organ systems, the absence of meaningful breed predisposition beyond the expected DSH majority, and the lack of district-level clustering, indicate a relatively uniform epidemiological landscape in which age and anatomical site are the principal determinants of risk.

Clinically, these findings argue for a low threshold for tissue diagnosis in older cats and systematic palpation/screening of mammary chains in females, supported by integrated diagnostic pathways. Continued, structured surveillance, leveraging and expanding national registries, will be essential to detect temporal shifts in incidence and case-mix and to translate epidemiological signal into earlier intervention and improved outcomes.

Overall, a coordinated approach that couples vigilant clinical screening with histopathological confirmation and complementary techniques offers the best prospect for timely diagnosis, tailored therapy, and improved prognostication in feline oncology.

## Figures and Tables

**Table 1:** Distribution of benign and malignant feline tumours according to anatomical location.

Anatomical Location	Benign		Malignant		Total
	*n*	%	*n*	%	*n*
Mammary	117	13.7%	735	86.3%	852
Cutaneous and Soft tissue	296	36.6%	512	63.4%	808
Gastrointestinal tract	0	0%	106	100%	106
Oral cavity	7	18.9%	30	81.1%	37
Respiratory system	0	0%	21	100%	21
Haemolymphatic system	1	5%	19	95%	20
Urinary system	0	0%	13	100%	13
Reproductive system	8	38.1%	13	61.9%	21
Ocular system	0	0%	13	100%	13
Musculoskeletal system	1	9.1%	10	90.9%	11
Body cavities	0	0%	1	100%	1
Neuroendocrine	0	0%	1	100%	1
Total	430	22.6%	1474	77.4%	1904

**Table 2:** Anatomical location: totals (*n*, %), main diagnoses (*n*, % of all tumours), sex distribution, and age descriptors.

Anatomical Location	Total (*n*; %)	Main Diagnoses (*n*; % of All Tumours)	Sex Distribution	Age (Years) Median (IQR)
Mammary	852 (44.8%)	Mammary carcinoma (729; 38.3%) Mammary adenoma (117; 6.1%) Mammary sarcoma (6; 0.3%)	F: 823 (96.6%) M: 29 (3.4%)	F: 11 (8–13) M: 11 (9–13)
Cutaneous and Soft tissue	808 (42.4%)	Fibrosarcoma (152; 8.0%) Squamous cell carcinoma (121; 6.4%) Mast cell tumour (90; 4.8%) Apocrine adenoma (67; 3.3%) Trichoblastoma (48; 2.5%) Haemangioma (45; 2.4%)	F: 381 (47.2%) M: 427 (52.9%)	F: 10 (7–12) M: 9 (7–12)
Gastrointestinal tract	106 (5.6%)	Lymphoma (65; 3.4%) Gastric adenocarcinoma (16; 0.8%) Intestinal adenocarcinoma (16; 0.8%)	F: 48 (45.3%) M: 58 (54.7%)	F: 10 (8–13) M: 11 (8–12)
Oral cavity	37 (1.9%)	Squamous cell carcinoma (19; 1.0%) Fibrosarcoma (7; 0.4%) Haemangioma (5; 0.3%)	F: 20 (54.1%) M: 17 (46.0%)	F: 13 (10–15) M: 12 (9–15)
Respiratory system	21 (1.1%)	Squamous cell carcinoma (15; 0.8%) Lymphoma (5; 0.3%) Fibrosarcoma (1; 0.1%)	F: 5 (23.8%) M: 16 (76.2%)	F: 12 (9–14) M: 11 (10–11)
Reproductive system	21 (1.1%)	Uterine adenocarcinoma (7; 0.4%) Leiomyosarcoma (4; 0.2%) Fibroma (2; 0.1%)	F: 20 (95.2%) M: 1 (4.8%)	F: 10 (7–13) M: 12 (single case)
Haemolymphatic system	20 (1.1%)	Lymphoma (14; 0.7%) Histiocytic sarcoma (2; 0.1%) Thymoma (2; 0.1%)	F: 8 (40%) M: 12 (60%)	F: 10 (4–14) M: 8 (6–12)
Urinary system	13 (0.7%)	Renal adenocarcinoma (5; 0.3%) Lymphoma (4; 0.2%) Urothelial carcinoma (4; 0.2%)	F: 5 (38.5%) M: 8 (61.5%)	F: 11 (5–14) M: 13 (12–15)
Ocular system	13 (0.7%)	Squamous cell carcinoma (11; 0.6%) Carcinoma (1; 0.1%) Melanoma (1; 0.1%)	F: 2 (15.4%) M: 11 (84.6%)	F: 9 (7–11) M: 8 (4–9)
Musculoskeletal system	11 (0.6%)	Osteosarcoma (6; 0.3%) Chondrosarcoma (3; 0.2%) Osteoma (1; 0.1%)	F: 8 (72.7%) M: 3 (27.3%)	F: 11 (9–14) M: 10 (10–12)
Body cavities	1 (0.1%)	Mesothelioma (1; 0.1%)	F: 0 (0.0%) M: 1 (100%)	M: 4 (single case)
Neuroendocrine	1 (0.1%)	Chemodectoma (1; 0.1%)	F: 0 (0.0%) M: 1 (100%)	M: 6 (single case)

## Data Availability

The data presented in this study are available on request from the corresponding author.

## Supplementary Materials

The following supporting information can be downloaded at https://www.mdpi.com/article/10.3390/ani16030364/s1. Supplementary Table S1. Feline tumours by anatomical location and diagnosis: totals (*n*, % of all cases) and within-location diagnosis (*n*, %).

## References

[1] Cannon C.M. (2015). Cats, Cancer and Comparative Oncology. Vet. Sci..

[2] Pinello K., Amorim I., Pires I., Canadas-Sousa A., Catarino J., Faísca P., Branco S., Peleteiro M.C., Silva D., Severo M. (2022). Vet-OncoNet: Malignancy Analysis of Neoplasms in Dogs and Cats. Vet. Sci..

[3] Giugliano R., Dell’Anno F., De Paolis L., Crescio M.I., Ciccotelli V., Vivaldi B., Razzuoli E. (2024). Mammary gland, skin and soft tissue tumors in pet cats: Findings of the feline tumors collected from 2002 to 2022. Front. Vet. Sci..

[4] Huber D., Severin K., Vlahović D., Križanac S., Mofardin S., Buhin I.M., Zagradišnik L.M., Šoštarić-Zuckermann I.-C., Kurilj A.G., Artuković B. (2024). Cancer morbidity in Croatian cats: Retrospective study on spontaneously arising tumors (2009–2019). Top. Companion Anim. Med..

[5] Di Teodoro G., Cito F., Salini R., Baffoni M., Defourny S.V.P., Cocco A., D’Alterio N., Palmieri C., Petrini A. (2024). Pathology-Based Animal Cancer Registry of Abruzzo and Molise Regions (Central Italy): A Ten-Year Retrospective Study (2014–2023). Vet. Sci..

[6] Seung B.-J., Bae M.-K., Sur J.-H. (2024). Regional Variations in and Key Predictors of Feline Tumor Malignancy: A Decade-Long Retrospective Study in Korea. Animals.

[7] Negoescu A., Borfalău C.-D., Gal C., Taulescu M., Catoi C. (2025). Epidemiology of gastrointestinal proliferative neoplastic-like lesions and tumors in dogs and cats: A retrospective study in two Romanian reference laboratories. Cluj Vet. J..

[8] Srisawat W., Pringproa K., Prachasilchai W., Thongtharb A., Sthitmatee N. (2024). Epidemiology and classification for canine and feline mammary gland tumors: A histopathological survey of 437 mammary gland tumor biopsies performed in a secondary care hospital in Chiang Mai, Thailand from 2012 to 2019. PeerJ.

[9] Tunç A.S., Filikçi K., Sağlam M., Kutsal O. (2023). Primary bone tumors in dogs and cats: 98 cases. Vet. Hekimler Derneği Derg..

[10] Laissaoui N., Simon Betz D., Millán Y., El Mrini M., Azrib R., Tligui N. (2024). Common tumors in cats: A comprehensive review of epidemiological and clinicopathological aspects. Vet. Integr. Sci..

[11] Dorn C., Taylor D., Frye F., Hibbard H. (1968). Survey of animal neoplasms in Alameda and Contra Costa Counties, California. I. Methodology and description of cases. J. Natl. Cancer Inst..

[12] MacVean D.W., Monlux A.W., Anderson P.S., Silberg S.L., Roszel J.F. (1978). Frequency of canine and feline tumors in a defined population. Vet. Pathol..

[13] Vascellari M., Baioni E., Ru G., Carminato A., Mutinelli F. (2009). Animal tumour registry of two provinces in northern Italy: Incidence of spontaneous tumours in dogs and cats. BMC Vet. Res..

[14] Manuali E., Forte C., Vichi G., Genovese D.A., Mancini D., De Leo A.A.P., Cavicchioli L., Pierucci P., Zappulli V. (2020). Tumours in European Shorthair cats: A retrospective study of 680 cases. J. Feline Med. Surg..

[15] Egenvall A., Nødtvedt A., Häggström J., Ström Holst B., Möller L., Bonnett B.N. (2009). Mortality of life-insured Swedish cats during 1999–2006: Age, breed, sex, and diagnosis. J. Vet. Intern. Med..

[16] Shida T., Yamada T., Maruo T., Ishida T., Kawamura H., Takeda H., Sugiyama H., Ishikawa T., Ito T., Madarame H. (2010). A Retrospective Study in 1,070 Feline Tumor Cases of Japan. J. Jpn. Vet. Cancer Soc..

[17] Zambelli A. (2015). Feline Cancer Prevalence in South Africa (1998–2005): Contrasts with the Rest of the World. J. Basic Appl. Sci..

[18] Graf R., Grüntzig K., Hässig M., Axhausen K.W., Fabrikant S., Welle M., Meier D., Guscetti F., Folkers G., Otto V. (2015). Swiss Feline Cancer Registry: A Retrospective Study of the Occurrence of Tumours in Cats in Switzerland from 1965 to 2008. J. Comp. Pathol..

[19] Graf R., Grüntzig K., Boo G., Hässig M., Axhausen K.W., Fabrikant S., Welle M., Meier D., Guscetti F., Folkers G. (2016). Swiss Feline Cancer Registry 1965–2008: The Influence of Sex, Breed and Age on Tumour Types and Tumour Locations. J. Comp. Pathol..

[20] Pérez-Enriquez J.M., Romero-Romero L., Alonso-Morales R., Fuentes-Pananá E. (2020). Tumor prevalence in cats: Experience from a reference diagnostic center in Mexico City (2006–2018). Vet. México OA.

[21] Vilmis D., Melikova J., Chechneva A. (2024). Retrospective analysis of data on the prevalence of malignant neoplasms in dogs and cats. Agrar. Sci..

[22] Siripoonsub J., Sirivisoot S., Techangamsuwan S., Rungsipipat A. (2024). Histopathological patterns and immunophenotyping of feline lymphomas and incidence in Metropolitan Bangkok, Thailand. Vet. World.

[23] Ho N.T., Smith K.C., Dobromylskyj M.J. (2018). Retrospective study of more than 9000 feline cutaneous tumours in the UK: 2006–2013. J. Feline Med. Surg..

[24] Soares M., Marques C., Catarino J., Batista M., Catita J., Faísca P. (2021). National survey of cat tumors in 2019: A retrospective study. Rev. Lusófona Ciência Med. Veterinária.

[25] Pinello K., Pires I., Castro A.F., Carvalho P.T., Santos A., de Matos A., Queiroga F., Canadas-Sousa A., Dias-Pereira P., Catarino J. (2022). Cross Species Analysis and Comparison of Tumors in Dogs and Cats, by Age, Sex, Topography and Main Morphologies. Data from Vet-OncoNet. Vet. Sci..

[26] Ludwig L., Dobromylskyj M., Wood G., Van Der Weyden L. (2022). Feline Oncogenomics: What Do We Know about the Genetics of Cancer in Domestic Cats?. Vet. Sci..

[27] Schiffman J.D., Breen M. (2015). Comparative oncology: What dogs and other species can teach us about humans with cancer. Philos. Trans. R. Soc. Lond. B Biol. Sci..

[28] Zaki F., Hurvitz A. (1976). Spontaneous neoplasms of the central nervous system of the cat. J. Small Anim. Pract..

[29] Rissi D. (2023). A review of primary central nervous system neoplasms of cats. Vet. Pathol..

[30] Stebbins K., Morse C., Goldschmidt M. (1989). Feline Oral Neoplasia: A Ten-Year Survey. Vet. Pathol..

[31] Hayes A.M., Adams V.J., Scase T.J., Murphy S. (2007). Survival of 54 cats with oral squamous cell carcinoma in United Kingdom general practice. J. Small Anim. Pract..

[32] Cray M., Selmic L.E., Ruple A. (2020). Demographics of dogs and cats with oral tumors presenting to teaching hospitals: 1996–2017. J. Vet. Sci..

[33] Brilhante-Simões P., Delgado L., Martins Â., Silva A., Monteiro L., Marcos R., Prada J. (2025). Association Between Cytological and Histopathological Diagnoses of Neoplastic and Non-Neoplastic Lesions in Oral Cavity from Dogs and Cats: An Observational Retrospective Study of 103 Cases. Vet. Sci..

[34] Rissetto K., Villamil J.A., Selting K.A., Tyler J., Henry C.J. (2011). Recent Trends in Feline Intestinal Neoplasia: An Epidemiologic Study of 1,129 Cases in the Veterinary Medical Database from 1964 to 2004. J. Am. Anim. Hosp. Assoc..

[35] Oh J.H., Cho J.-Y. (2023). Comparative oncology: Overcoming human cancer through companion animal studies. Exp. Mol. Med..

[36] Rolph K., Cavanaugh R. (2022). Infectious Causes of Neoplasia in the Domestic Cat. Vet. Sci..

[37] Munday J., Thomson N., Henderson G., Fairley R., Orbell G. (2018). Identification of *Felis catus* papillomavirus 3 in skin neoplasms from four cats. J. Vet. Diagn. Investig..

[38] Mills S.W., Musil K.M., Davies J.L., Hendrick S., Duncan C., Jackson M.L., Kidney B., Philibert H., Wobeser B.K., Simko E. (2015). Prognostic Value of Histologic Grading for Feline Mammary Carcinoma:A Retrospective Survival Analysis. Vet. Pathol..

[39] De Campos C., Damasceno K., Gamba C., Ribeiro A., Machado C., Lavalle G., Cassali G. (2016). Evaluation of prognostic factors and survival rates in malignant feline mammary gland neoplasms. J. Feline Med. Surg..

[40] Burrai G.P., Baldassarre V., Brunetti B., Iussich S., Maniscalco L., Mariotti F., Sfacteria A., Cocumelli C., Grieco V., Millanta F. (2022). Canine and feline in situ mammary carcinoma: A comparative review. Vet. Pathol..

[41] Pickard Price P., Stell A., O’Neill D., Church D., Brodbelt D. (2023). Epidemiology and risk factors for mammary tumours in female cats. J. Small Anim. Pract..

[42] Simeonov R., Grozeva I. (2024). Epidemiological retrospective studies of feline mammary gland tumours in Bulgaria. Bulg. J. Vet. Med..

[43] Souza F., Moreira I., Dariva A., Nakagaki K., Abreu C., Balabram D., Cassali G. (2024). Epidemiologic and Clinicopathological Characterization of Feline Mammary Lesions. Vet. Sci..

[44] Monteiro M., Petrucci G., Queiroga F.L. (2025). Prognostic Insights in Feline Mammary Carcinomas: Clinicopathological Factors and the Proposal of a New Staging System. Animals.

[45] Miller M.A., Nelson S.L., Turk J.R., Pace L.W., Brown T.P., Shaw D.P., Fischer J.R., Gosser H.S. (1991). Cutaneous neoplasia in 340 cats. Vet. Pathol..

[46] Van der Linde-Sipman J.S., de Wit M.M.L., van Garderen E., Molenbeek R.F., van der Velde-Zimmermann D., de Weger R.A. (1997). Cutaneous Malignant Melanomas in 57 Cats: Identification of (Amelanotic) Signet-ring and Balloon Cell Types and Verification of Their Origin by Immunohistochemistry, Electron Microscopy, and In Situ Hybridization. Vet. Pathol..

[47] Hirayama K., Endoh C., Kagawa Y., Ohmachi T., Yamagami T., Nomura K., Matsuda K., Okamoto M., Taniyama H. (2017). Amyloid-Producing Odontogenic Tumors of the Facial Skin in Three Cats. Vet. Pathol..

[48] Stoll A., Suárez-Bonnet A., Summers B., Priestnall S. (2018). Malignant Cutaneous Peripheral Nerve Sheath Tumour with Rhabdomyosarcomatous Differentiation (Triton Tumour) in a Domestic Cat. J. Comp. Pathol..

[49] Jung J.-Y., Ko K.-R., Choi Y.-M., Jang S.-H., Kim J.-H. (2019). A retrospective study of feline cutaneous tumors in Korea from 2013 to 2018. Korean J. Vet. Res..

[50] Filgueira K., Chalita M., Sellera F., Reche-Júnior A. (2022). Cytopathology of cutaneous and subcutaneous neoplasms in feline species: A retrospective study. Acta Vet. Bras..

[51] Dobromylskyj M. (2022). Feline Soft Tissue Sarcomas: A Review of the Classification and Histological Grading, with Comparison to Human and Canine. Animals.

[52] Bertone E.R., Snyder L.A., Moore A.S. (2003). Environmental and lifestyle risk factors for oral squamous cell carcinoma in domestic cats. J. Vet. Intern. Med..

[53] Murphy S. (2013). Cutaneous squamous cell carcinoma in the cat: Current understanding and treatment approaches. J. Feline Med. Surg..

[54] Pellin M., Turek M. (2016). A Review of feline oral squamous cell carcinoma. Today Vet. Pract..

[55] McGrath A., Chen C., Abrams B., Hixon L., Grimes J., Viani E., McLoughlin M., Tremolada G., Lapsley J., Selmic L. (2022). Clinical presentation and outcome in cats with aural squamous cell carcinoma: A review of 25 cases (2010–2021). J. Feline Med. Surg..

[56] Sequeira I., Pires M., Leitão J., Henriques J., Viegas C., Requicha J. (2022). Feline Oral Squamous Cell Carcinoma: A Critical Review of Etiologic Factors. Vet. Sci..

[57] Zaccone R., Renzi A., Chalfon C., Lenzi J., Bellei E., Marconato L., Ros E., Rigillo A., Bettini G., Faroni E. (2022). Environmental risk factors for the development of oral squamous cell carcinoma in cats. J. Vet. Intern. Med..

[58] Falcão F., Faísca P., Viegas I., de Oliveira J.T., Requicha J.F. (2020). Feline oral cavity lesions diagnosed by histopathology: A 6-year retrospective study in Portugal. J. Feline Med. Surg..

[59] Dean R.S., Pfeiffer D.U., Adams V.J. (2013). The incidence of feline injection site sarcomas in the United Kingdom. BMC Vet. Res..

[60] Cecco B.S., Henker L.C., De Lorenzo C., Schwertz C.I., Bianchi R.M., da Costa F.V.A., Driemeier D., Pavarini S.P., Sonne L. (2019). Epidemiological and Pathological Characterization of Feline Injection Site Sarcomas in Southern Brazil. J. Comp. Pathol..

[61] White M.E., Yang C., Hokamp J.A., Wellman M.L. (2020). Fibrosarcoma with sarcomatosis and metastasis in a FeLV-negative cat. Vet. Clin. Pathol..

[62] Cid G., Jardim M., Fernandes M., Bastos P., Santos C., Silva M., Nogueira V., Souza H. (2022). Primary Intestinal Fibrosarcoma in Cats. Acta Sci. Vet..

[63] Doddy F.D., Glickman L.T., Glickman N.W., Janovitz E.B. (1996). Feline fibrosarcomas at vaccination sites and non-vaccination sites. J. Comp. Pathol..

[64] Porcellato I., Menchetti L., Brachelente C., Sforna M., Reginato A., Lepri E., Mechelli L. (2017). Feline Injection-Site Sarcoma:Matrix Remodeling and Prognosis. Vet. Pathol..

[65] Bertone E.R., Snyder L.A., Moore A.S. (2002). Environmental tobacco smoke and risk of malignant lymphoma in pet cats. Am. J. Epidemiol..

[66] Wilson H.M. (2008). Feline Alimentary Lymphoma: Demystifying the Enigma. Top. Companion Anim. Med..

[67] Ku C.K., Kass P.H., Christopher M.M. (2017). Cytologic-histologic concordance in the diagnosis of neoplasia in canine and feline lymph nodes: A retrospective study of 367 cases. Vet. Comp. Oncol..

[68] Almendros A., Chan L.-K., dos Santos Horta R., Nekouei O., Hill F., Giuliano A. (2024). Description and Characterization of Different Types of Lymphoma in Cats in Hong Kong. Animals.

[69] Moore P.F., Rodriguez-Bertos A., Kass P.H. (2012). Feline Gastrointestinal Lymphoma:Mucosal Architecture, Immunophenotype, and Molecular Clonality. Vet. Pathol..

[70] Leite-Filho R.V., Panziera W., Bandinelli M.B., Henker L.C., da Conceição Monteiro K., Corbellini L.G., Driemeier D., Sonne L., Pavarini S.P. (2020). Epidemiological, pathological and immunohistochemical aspects of 125 cases of feline lymphoma in Southern Brazil. Vet. Comp. Oncol..

[71] Bennett P., Williamson P., Taylor R. (2024). Demographics of Feline Lymphoma in Australian Cat Populations: 1705 Cases. Vet. Sci..

[72] Lo Giudice A., Porcellato I., Giglia G., Sforna M., Lepri E., Mandara M.T., Leonardi L., Mechelli L., Brachelente C. (2024). Exploring the Epidemiology of Melanocytic Tumors in Canine and Feline Populations: A Comprehensive Analysis of Diagnostic Records from a Single Pathology Institution in Italy. Vet. Sci..

[73] Pittaway R., Dobromylskyj M., Erles K., Pittaway C., Suárez-Bonnet A., Chang Y.-M., Priestnall S. (2019). Nonocular Melanocytic Neoplasia in Cats: Characterization and Proposal of a Histologic Classification Scheme to More Accurately Predict Clinical Outcome. Vet. Pathol..

[74] Isaza D., Robinson N., Pizzirani S., Pumphrey S. (2020). Evaluation of cytology and histopathology for the diagnosis of feline orbital neoplasia: 81 cases (2004–2019) and review of the literature. Vet. Ophthalmol..

[75] Jones B., Cotterill N., Drees R., Dietrich U., Purzycka K. (2022). Tumours involving the retrobulbar space in cats: 37 cases. J. Feline Med. Surg..

[76] Kayes D., Blacklock B. (2022). Feline Uveal Melanoma Review: Our Current Understanding and Recent Research Advances. Vet. Sci..

[77] Cecco B., Silva T., Felin D., Weber V.M., Dutra L., Kommers G., Andrade C. (2018). Malignant peripheral nerve sheath tumor in a cat: Cytological, histopathological, and immunohistochemical aspects. Comp. Clin. Pathol..

[78] De Cecco B., Argenta F., Bianchi R., De Lorenzo C., Wronski J., Bandinelli M., Da Costa F.V., Driemeier D., Pavarini S., Sonne L. (2020). Feline giant-cell pleomorphic sarcoma: Cytologic, histologic and immunohistochemical characterization. J. Feline Med. Surg..

[79] Van Sprundel R., Van Den Ingh T., Guscetti F., Kershaw O., Van Wolferen M., Rothuizen J., Spee B. (2014). Classification of primary hepatic tumours in the cat. Vet. J..

[80] Davis-Thompson Foundation World Health Organization Tumor Fascicles. https://davisthompsonfoundation.org/product-category/world-health-organization-tumor-fascicles/.

[81] Villamil J.A., Henry C.J., Bryan J.N., Ellersieck M., Schultz L., Tyler J.W., Hahn A.W. (2011). Identification of the most common cutaneous neoplasms in dogs and evaluation of breed and age distributions for selected neoplasms. J. Am. Vet. Med. Assoc..

[82] Brilhante-Simões P., Lopes R., Delgado L., Machado A., Silva A., Martins Â., Marcos R., Queiroga F., Prada J. (2025). What Comes from Cytology Diagnosis: A Comprehensive Epidemiological Retrospective Analysis of 3068 Feline Cases. Vet. Sci..

[83] Petrie A., Watson P.F. (2013). Statistics for Veterinary and Animal Science.

[84] Goecke H., Hüther M. (2016). Regional Convergence in Europe. Intereconomics.

[85] Rodríguez-Pose A., Ketterer T. (2020). Institutional change and the development of lagging regions in Europe. Reg. Stud..

[86] Bergamini E., Zachmann G. (2021). Exploring EU’s Regional Potential in Low-Carbon Technologies. Sustainability.

[87] Guerreiro G., Rego C. (2005). Regional Delimitation in Continental Portugal: What Does Cluster Analysis Tell Us?.

[88] Sokołowski A., Markowska M., Strahl D., Sobolewski M. (2015). The Influence of Upper Level NUTS on Lower Level Classification of EU Regions. Proceedings of the Data Science, Learning by Latent Structures, and Knowledge Discovery.

[89] Rodrigues-Jesus J., Vilhena H., Canadas-Sousa A., Dias-Pereira P. (2025). Feline Mammary Tumors: A Comprehensive Review of Histological Classification Schemes, Grading Systems, and Prognostic Factors. Vet. Sci..

[90] Saba C.F. (2017). Vaccine-associated feline sarcoma: Current perspectives. Vet. Med..

[91] Overley B., Shofer F., Goldschmidt M., Sherer D., Sorenmo K. (2005). Association between ovarihysterectomy and feline mammary carcinoma. J. Vet. Intern. Med..

[92] Țuțu P., Tanase I.O., Dascalu M., Pasca A., Hritcu O., Soreanu O., Bocaneti F.D., Mares M. (2024). Histopathological Caracterization of Skin and Oral Lesions in Domestic Cats (*Felis catus*). Sci. Pap. J. Vet. Ser..

