# Supplementary material for: Mapping Feline Oncology in Portugal: A National Characterization
Source: Animals (Basel). 2026 Jan 23;16(3):364. doi: 10.3390/ani16030364 (PMC12897375; doi:10.3390/ani16030364)
Supplement: Supplementary file 1 [file animals-16-00364-s001.zip › animals-4037216-supplementary.pdf]

Supplementary Table S1. Feline tumours by anatomical location and diagnosis: totals (*n*, % of all cases) and within-location diagnosis (*n*, % per location).

Anatomical Location	Total (*n*; %)	Within-location Diagnosis (*n*; % per location)
Mammary	852 (44.8%)	Tubular carcinoma (232; 27.2%)
		Tubulopapillary carcinoma (182; 21.4%)
		Papillary carcinoma (83; 9.7%)
		Fibroadenoma (82; 9.6%)
		Cribiform carcinoma (52; 6.1%)
		Ductal carcinoma (51; 6.0%)
		Comedocarcinoma (49; 5.8%)
		Solid carcinoma (39; 4.6%)
		Carcinoma in situ (29; 3.4%)
		Papillary adenoma (21; 2.5%)
		Tubular adenoma (12; 1.4%)
		Micropapillary carcinoma (7; 0.8%)
		Carcinosarcoma (3; 0.4%)
		Adenosquamous carcinoma (3; 0.4%)
		Fibrosarcoma (2; 0.2%)
		Tubulopapillary adenoma (1; 0.1%)
		Mucinous carcinoma (1; 0.1%)
		Intraductal carcinoma (1; 0.1%)
		Haemangiosarcoma (1; 0.1%)
		Complex adenoma (1; 0.1%)
Cutaneous and Soft tissue	808 (42.4%)	Fibrosarcoma (152; 18.8%)
		Squamous cell carcinoma (121; 15.0%)
		Mast cell tumour (90; 11.1%)
		Apocrine adenoma (67; 8.3%)
		Trichoblastoma (48; 5.9%)
		Haemangioma (45; 5.6%)
		Basal cell tumour (43; 5.3%)
		Lipoma (30; 3.7%)
		Haemangiosarcoma (29; 3.6%)
		Nerve sheath tumour (25; 3.1%)
		Melanoma (22; 2.7%)
		Ceruminous adenocarcinoma (18; 2.2%)
		Apocrine adenocarcinoma (16; 2.0%)
		Sarcoma (14; 1.7%)
		Squamous cell carcinoma in situ (13; 1.6%)
		Myxosarcoma (8; 1.0%)
		Adenosquamous carcinoma (7; 0.9%)
		Basal cell carcinoma (7; 0.9%)
		Apocrine cystadenoma (6; 0.7%)
		Myxoma (5; 0.6%)
		Ceruminous adenoma (4; 0.5%)
		Lymphoma (4; 0.5%)
		Sebaceous adenoma (4; 0.5%)
		Anaplastic giant cell sarcoma (2; 0.3%)
		Ductal adenoma (2; 0.3%)
		Fibroma (2; 0.3%)
		Liposarcoma (2; 0.3%)
		Lymphangioma (2; 0.3%)
		Squamous papilloma (2; 0.3%)
		Trichofolliculoma (2; 0.3%)
		Anaplastic sarcoma (1; 0.1%)
		Angiolipoma (1; 0.1%)
		Basosquamous carcinoma (1; 0.1%)

		Cholesteatoma (1; 0.1%)<br>Dermatofibroma (1; 0.1%)<br>Epitelioma (1; 0.1%)<br>Fibrolipoma (1; 0.1%)<br>Hamartoma (1; 0.1%)<br>Lymphangiosarcoma (1; 0.1%)<br>Melanocytoma (1; 0.1%)<br>Osteoma (1; 0.1%)<br>Perivascular wall tumor (1; 0.1%)<br>Rhabdomyosarcoma (1; 0.1%)<br>Sebaceous carcinoma (1; 0.1%)<br>Trichoepithelioma (1; 0.1%)<br>Tricholemmoma (1; 0.1%)
Gastrointestinal tract	106 (5.6%)	Lymphoma (65; 61.3%)<br>Gastric adenocarcinoma (16; 15.1%)<br>Intestinal adenocarcinoma (16; 15.1%)<br>Leiomyosarcoma (2; 1.9%)<br>Salivary adenocarcinoma (2; 1.9%)<br>Colangiocarcinoma (1; 0.9%)<br>Gallbladder carcinoma (1; 0.9%)<br>Gastrointestinal stromal tumour (1; 0.9%)<br>Hepatocelular carcinoma (1; 0.9%)<br>Mast cell tumour (1; 0.9%)
Oral cavity	37 (1.9%)	Squamous cell carcinoma (19; 51.4%)<br>Fibrosarcoma (7; 18.9%)<br>Haemangioma (5; 13.5%)<br>Lymphoma (3; 8.1%)<br>Ameloblastoma (1; 2.7%)<br>Fibromatous epulis (1; 2.7%)<br>Melanoma (1; 2.7%)
Respiratory system	21 (1.1%)	Squamous cell carcinoma (15; 71.4%)<br>Lymphoma (5; 23.8%)<br>Fibrosarcoma (1; 4.8%)
Reproductive system	21 (1.1%)	Uterine adenocarcinoma (7; 33.3%)<br>Leiomyosarcoma (4; 19.1%)<br>Fibroma (2; 9.5%)<br>Granulosa cell tumour (2; 9.5%)<br>Leiomyoma (2; 9.5%)<br>Malignant Sertoli cell tumor (1; 4.8%)<br>Ovarian carcinoma (1; 4.8%)<br>Teratoma (1; 4.8%)<br>Thecal cell tumour (1; 4.8%)
Haemolymphatic system	20 (1.1%)	Lymphoma (14; 70%)<br>Histiocytic sarcoma (2; 10%)<br>Thymoma (2; 10%)<br>Haemophagocytic histiocytic sarcoma (1; 5%)<br>Haemangiosarcoma (1; 5%)
Urinary system	13 (0.7%)	Renal adenocarcinoma (5; 38.5%)<br>Lymphoma (4; 30.8%)<br>Urothelial carcinoma (4; 30.8%)
Ocular system	13 (0.7%)	Squamous cell carcinoma (11; 84.6%)<br>Carcinoma (1; 7.7%)<br>Melanoma (1; 7.7%)
Musculoskeletal system	11 (0.6%)	Osteosarcoma (6; 54.6%)<br>Chondrosarcoma (3; 27.3%)<br>Osteoma (1; 9.1%)<br>Squamous cell carcinoma (1; 9.1%)

Body cavities	1 (0.1%)	Mesothelioma (1; 100%)
Neuroendocrine	1 (0.1%)	Chemodectoma (1; 100%)
